# Evaluation of the skeletal and dental effects of a hybrid aesthetic functional appliance (HAF) in skeletal class II division 1 malocclusion: A prospective uncontrolled clinical trial

**DOI:** 10.34172/joddd.40732

**Published:** 2024-03-29

**Authors:** Sohaila Adnan Ahmed Zehairy, Mohammad Hasan Abdellatief, Ahmed Maher Fouda

**Affiliations:** ^1^Faculty of Dentistry, Mansoura University, Mansoura, Egypt; ^2^Orthodontics Department, Faculty of Dentistry, Mansoura University, Mansoura, Egypt

**Keywords:** Aesthetic, Cl II malocclusion, Deficiency, Functional appliance, HAF, Hybrid, Mandible

## Abstract

**Background.:**

The present study investigated the skeletal and dental effect in class II division I growing patients due to mandibular deficiency treated with the hybrid aesthetic functional (HAF) appliance.

**Methods.:**

A sample of 16 growing patients (5 boys and 11 girls; mean age: 9.50 years, standard deviation: 1.15) with class II division I malocclusion were treated using the HAF appliance for an average period of 10±3 months. For each patient, a cephalometric radiograph was taken before and after treatment, and digital analysis was applied using the WebCeph program. The statistical analysis was performed to evaluate dental and skeletal changes associated with the HAF appliance and determine if there were any statistically significant variations in anatomical measurements between the start and completion of the treatment.

**Results.:**

The data showed a significant increase in SNB angle (*P*=0.002), leading to a significant decrease in ANB angle (*P*=0.001). The mandibular length significantly increased (*P*=0.008), the lower incisors were flared significantly (*P*=0.028), and the lower molars were extruded significantly (*P*≤0.001). Also, this study revealed a significant decrease in Wits appraisal (*P*≤0.001), overjet (*P*≤0.001), and overbite (*P*=0.041). Additionally, a significant increase in lower anterior facial height (*P*≤0.001), total facial height (*P*=0.001), and posterior facial height (*P*=0.037) were observed.

**Conclusion.:**

The HAF appliance showed that it could be used to correct class II division 1 skeletal discrepancy by mandibular advancement. The HAF appliance increased all facial heights significantly.

## Introduction

 Class II malocclusion is one of the most prevalent forms of malocclusion observed in ordinary orthodontic practice.^1^ Class II skeletal malocclusion may result from retruded mandibular growth, protruded maxillary growth, or a combination of the two.^2^ According to McNamara, it is uncommon to find skeletal protrusion of the maxilla. Therefore, it seems that when developing the best course of action, treatments that alter the rate and pattern of mandibular growth are often more appropriate than those that impede maxillary development.^3^

 The functional appliances enhance mandibular development by advancing the position of the mandible and correcting skeletal and occlusal disharmony. Dentoalveolar and skeletal effects are produced by functional appliances used in the early correction of Class II patients. Retraction of the upper incisors and flaring of the lower incisors are signs of dentoalveolar effects, while the remodeling of the glenoid fossa, accelerated condyle development, and neuromuscular adaptation are signs of skeletal impacts.^4^ Based on a systematic review, removable functional appliances have fewer skeletal effects on growing patients than untreated ones. Therefore, treating Class II malocclusion with removable functional appliances has significant dentoalveolar effects while only mildly stimulating mandibular growth and somewhat restricting maxillary growth.^5^

 Additionally, functional appliances used to treat growing individuals with substantial overjet decrease the possibility of needing orthognathic surgery. However, the patient’s cooperation is essential for the treatment’s effectiveness using a removable functional appliance.

 Since the development of the activator appliance by Andersen, the popularity of functional appliances to treat class II malocclusion has increased. The activator and twin block are the most commonly used orthopedic functional appliances for class II skeletal malocclusions. Patients were embarrassed to wear them in public because they are made from unattractive metal and acrylic elements.

**Figure 1:**
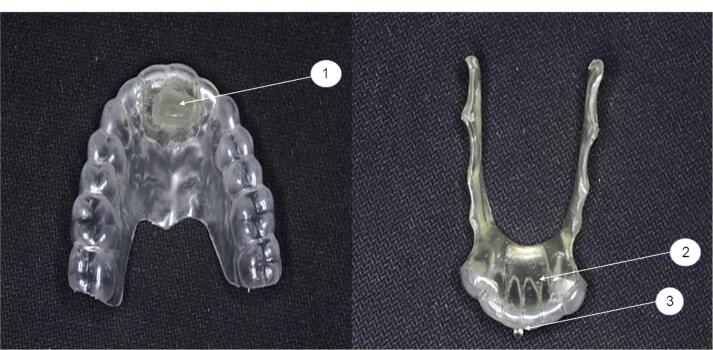
The HAF appliance with a double-plate design with thermoplastic and acrylic components (1) Advancement bar. (2) Guiding surface. (3) Buttons

 Unquestionably, the increasing need for visually enhanced treatment options is the driving force behind modern orthodontic technology. Following this trend, patients seek alternative orthodontic solutions, like clear aligners and lingual appliances, which are more appealing than various forms of buccal permanent attachments.^6-8^ Recently, preteen and adolescent patients’ aesthetic needs have expanded enormously. Children can differentiate between different sorts of grins as early as the age of five, and by the age of eight, the aesthetic canons of adults are well-established.^8^ Efforts have been made to improve the comfort and aesthetic of removable appliances to boost patient compliance with removable appliances. The hybrid aesthetic functional (HAF) appliance was designed by Dr. Christos Livas (Figure 1) to integrate aesthetics and orthodontic function.^9^ This study evaluated the skeletal and dental effects of HAF appliance, which might bridge the aesthetic desire of young patients with traditional dentofacial orthopedics.

## Methods

###  Study population

 This study included 16 participants (5 boys and 11 girls with a mean age of 9.50 years; standard deviation: 1.15). They were recruited from the Orthodontics Department of Mansoura University’s Faculty of Dentistry. The Ethics Committee of Mansoura University approved this study’s protocol (A05051021). The eligibility criteria included age factor in the mixed dentition stage (8‒12 years), skeletal Class II division 1 malocclusion due to mandibular deficiency, overjet exceeding 4 mm, no orthodontic treatment in the past, good oral hygiene, and no congenital craniofacial deformity or systemic disease.

###  Appliance design and treatment protocol (interventions)

 The HAF appliance has a double-plate design with thermoplastic and acrylic components (Figure 1). The upper part is a vacuum-formed plate composed of a 2-mm-thick transparent hard elastic polyethylene sheet (Easy-Vac Gasket, Korea), which covers all the upper teeth and an acrylic advancement bar positioned anteriorly in the midpalatal area.

 The lower part is made from a 2-mm-thick transparent hard elastic polyethylene sheet (Easy-Vac Gasket, Korea) that completely covers the lower six anterior teeth. An acrylic guide surface is applied to suit the upper advancement bar on the lingual surfaces of the lower anterior teeth. In accordance with the lingual vestibule’s morphology, two-sided arms are stretched from the acrylic resin body to the second molars. A white acrylic resin was used during the appliance fabrication to improve the aesthetics of its acrylic parts. Acrylic buttons were made at the buccal cervical thirds of the upper and lower dental midlines.

 Before beginning therapy, the patient’s guardians were told about the study and signed a consent form. The study began in December 2021 and ended in February 2023. First, the records were made for each patient, including photos (extraoral and intraoral), study casts, and wrist, panoramic, and cephalometric radiographs. Diagnostic study sheets customized by the Department of Orthodontics were used to collect pretreatment information from the patients’ dental and medical history and clinical examinations.

 Following a check of the amount of overjet using a Polyguage, the amount of sagittal advancement was accomplished gradually with no more than 6 mm per advancement, guided by an Exacto-Bite stick to prevent or decrease pain during treatment.^10^ A vertical separation from posterior area (about 5 mm) was established to allow lower posterior teeth to erupt and about 2 mm vertical separation in anterior area. The advanced bite registration was obtained by a horseshoe-shaped wafer of medium-hard wax between the maxillary and mandibular arches in the centric occlusion using the Exacto-Bite stick. The midline of the upper and lower arches was evaluated in centric relation. Two impressions were made: one for the study cast and the other for manufacturing the HAF appliance.

**Figure 2:**
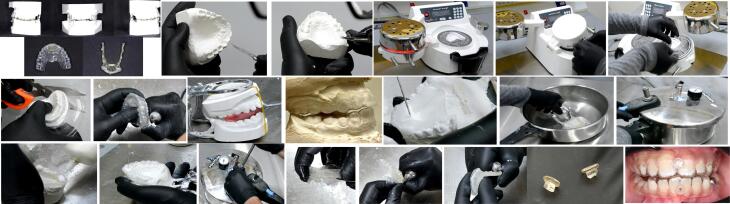
Fabrication process of appliances by an orthodontic laboratory

 All appliances were fabricated by a single orthodontic laboratory using the following steps (Figure 2). First, the undercuts were relieved on the cast. The compact pressure molding unit (MINISTAR S, Germany) was used to compress the polyethylene sheets. The excess thermoplastic material was cut with scissors, and the appliance was finished and polished. The working casts were mounted with an Exacto-Bite on a simple hinge articulator. After that, the acrylic parts were added, beginning with the lower member to make the guiding surface and extension lingual arms. The cast and the appliance were immersed in water under pressure to eliminate extra air bubbles from the acrylic resin. Then, the lower member of the appliance was finished. On the other hand, the acrylic part was added to the upper member to build the advancement bar after adding a wax coating to the surface of the lower member. The appliance’s upper and lower members were placed in water under pressure. A straight contra was used to finish and polish the HAF appliance. The buttons were constructed from white acrylic resin and added to the appliance using self-cured acrylic resin.

###  Appliance delivery and patient instructions

**Figure 3:**
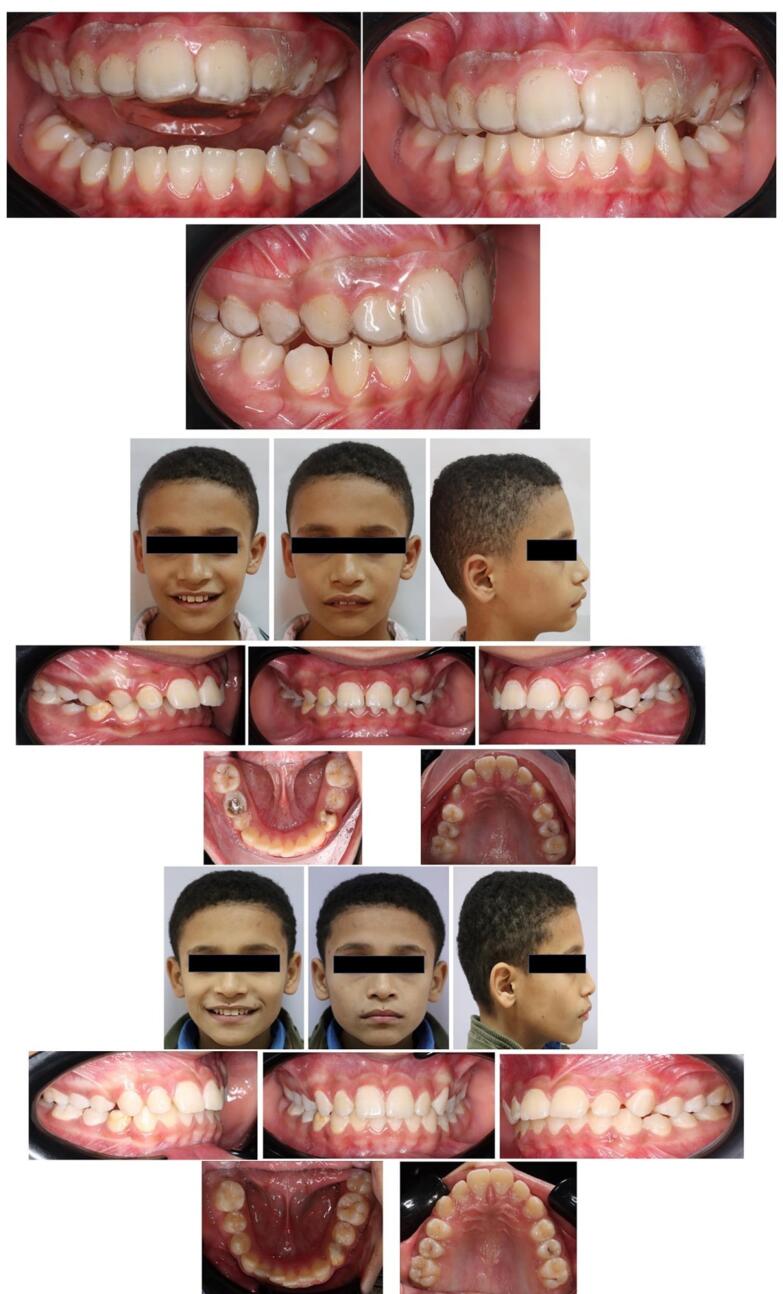
Clinical photographs of participants

 Inside the patient’s mouth, the upper and lower members of the appliance were inspected separately. Both members of the appliance were worn by the patient, who was asked to advance his mandible so the lower guiding surface could engage the upper advancement plane. The patient was instructed on proper appliance placement and removal, to wear the HAF appliance continuously except during eating time, to maintain proper oral hygiene by brushing his teeth after every meal, to keep regular appointments every three weeks, and to use intraoral vertical elastics between the upper and lower buttons at night to prevent upper and lower members disengagement. However, some patients eventually stopped using intermaxillary elastics as they found it difficult to maintain them in place while sleeping. Finally, when the overjet was rectified, and the molars achieved Cl I or super Cl I molar relation, the upper member of the appliance was kept as an anterior bite plane for retention (Figure 3).

###  Cephalometric analysis

 All the cephalometric radiographs were digitally traced, and all the measurements were made by the WebCeph program (WebCeph^TM^ is a Dental Imaging software, version 1.5.0, Assemble Circle Corp., the Republic of Korea). Before and after treatment, the following data were obtained to evaluate the dental and skeletal effects of the HAF appliance (Table 1-4).

**Table 1:** Maxillary skeletal and dentoalveolar measures

**SNA**	The angle formed by sella, nasion, and A
**ANS-PNS (mm)**	The distance from the anterior to the posterior nasal spine
**U1-SN (0)**	The angle created by the maxillary central incisor’s long axis and SN plane
**U1-PP (mm)**	The distance between the incisal edge of the central maxillary incisor and the palatal plane

**Table 2:** Mandibular skeletal and dentoalveolar measurements

**SNB (0)**	The angle created between the SN and NB planes
**FMA (0)**	The angle formed between the Frankfort and mandibular planes
**GoGn-SN (0)**	The angle between mandibular and SN planes
**Go-Gn (mm)**	Length of mandible
**Li-MP (0)**	The angle between the long axis of the central incisor of the mandible and the plane of the mandible
**L1-GnGo (mm)**	The distance between the incisal edge of the lower incisor and the plane of the mandible
**L6-GnGo (mm)**	The distance between the mesial cusp tip of the lower first molar and the plane of the mandible

**Table 3:** Maxillary mandibular measurements

**GoGn-SN (0)**	The angle between mandibular and SN planes
**ANB (0)**	The angle between the planes NA and NB (ANB = SNA - SNB)
**Wits (mm)**	The distance where the functional occlusal plane is intersected by two lines drawn from points A and B
**Overjet (mm)**	The horizontal distance between the lower incisors’ labial surfaces and the most prominent upper central incisor’s incisal tip
**Overbite (mm)**	The vertical overlap between the most prominent upper central incisor and the labial surface of the most prominent lower incisors
**Interincisal angle**	The angle formed between the long axis of the maxillary and mandibular central incisors

**Table 4:** Facial height measurements

**Posterior facial height (mm)**	The distance from sella to gonion
**Total anterior facial height (mm)**	The distance from nasion to menton
**Lower anterior facial height (mm)**	The distance from the anterior nasal spine to menton

###  Sample size calculation

 The PASS 15 Power Analysis and Sample Size Software (2017, NCSS, LLC. Kaysville, Utah, USA, ncss.com/software/pass) was used to determine the sample size. A sample size of 16 data pairs achieves 80.3% power to reject the null hypothesis of zero effect size when the population effect size is 1.00, and the significance level (alpha) is 0.050 using a two-sided paired *t *test.

###  Statistical analysis

 SPSS 24 (IBM, October 2009) for Windows was used to analyze the data. A Kolmogorov-Smirnov test was first used to test the normality of data. Numbers and percentages were used to describe the qualitative data. Continuous variables were shown as mean ± SD (standard deviation) for normally distributed. Quantitative factors were compared before and after using a paired *t *test. The 5% level was set as the significance criterion. The results were considered significant at *P* ≤ 0.05.

## Results

###  Demographic data

Table 5 presents the means and standard deviations (SD) for the distribution of the studied cases according to age. The ages of the subjects ranged from 8 to 12 years. The patients included five males and eleven females.

**Table 5:** Demographic data of the group under study

**Demographic data**	**The studied group (n=16)**
Age (years)	
Mean ± SD	9.50 ± 1.15
Min-Max	8.0-12.0
Gender	
Male	5 (31.2%)
Female	11 (68.8%)

###  Skeletal and dentoalveolar measurements of the maxilla

 Nonsignificant increases were detected in the SNA angle and ANS-PNS before and after treatment. U1-SN (0) and U1-PP (mm) did not show any discernible alterations (Table 6).

**Table 6:** Comparison of changes in maxillary skeletal and dentoalveolar measurements before and after treatment

**Maxillary skeletal**	**Before (n=16)**	**After (n=16)**	**Mean difference (95% CI)**	***P*** ** value**
SNA (0)	81.52 ± 2.87	81.59 ± 3.11	0.07 (-0.54‒0.67)	t = 0.241 *P* = 0.813
ANS-PNS (mm)	47.56 ± 2.24	48.60 ± 2.21	1.04 (-0.62‒2.7)	t = 1.332 *P* = 0.203
U1-SN (0).	109.64 ± 6.82	108.19 ± 7.69	-1.45 (-3.73‒0.83)	t = 1.35 *P* = 0.196
U1-PP (mm)	25.39 ± 3.67	25.60 ± 3.01	0.21 (-0.56‒0.98)	t = 0.567 *P* = 0.579

###  Skeletal and dentoalveolar measurements of the mandible

 Statistically significant increases in the SNB angle and length of the mandible were observed, while there was a nonsignificant increase in SN-MP and FMA angles. The Li-MP and L6-GnGo showed a statistically significant rise (*P* < 0.05), but the L1-GnGo (mm) exhibited no change (Table 7).

**Table 7:** Comparison of changes in skeletal and dentoalveolar measurements of the mandible before and after treatment

**Mandibular skeletal**	**Before (n=16)**	**After (n=16)**	**Mean difference (95% CI)**	***P*** ** value**
SNB (0)	74.85 ± 2.67	76.05 ± 3.29	1.19 (0.49‒1.90)	t = 3.62 *P* = 0.002*
SN-MP (0)	34.62 ± 5.53	35.28 ± 5.56	0.65 (0.54‒0.51)	t = 1.20 *P* = 0.248
FMA (0).	25.10 ± 4.48	26.79 ± 5.82	1.69 (-0.28‒3.67)	t = 1.82 *P* = 0.087
GnGo	62.02 ± 4.33	64.58 ± 4.32	2.56 (0.78‒4.33)	t = 3.07 *P* = 0.008*
L1-GnGo (mm)	32.13 ± 2.74	32.48 ± 2.30	0.36 (-0.42‒1.14)	t = 0.971 *P* = 0.347
Li-MP (0)	97.46 ± 7.34	100.41 ± 7.42	2.94 (0.35‒5.53)	t = 2.42 *P* = 0.028*
L6-GnGo (mm)	21.71 ± 2.43	23.35 ± 2.30	1.64 (1.13‒2.14)	t = 6.85 *P* ≤ 0.001*

* Statistically significant.

###  Maxillary and mandibular measurements

 All measurements (*P* < 0.05) indicated a substantial decrease, except for the interincisal angle, which showed no significant change (Table 8).

**Table 8:** Comparison of changes in maxillary and mandibular measurements before and after treatment

**Maxillary mandibular**	**Before (n=16)**	**After (n=16)**	**Mean difference (95% CI)**	***P*** ** value**
ANB Deg.	6.68 ± 1.78	5.54 ± 1.86	-1.13 (-1.7‒ -0.56)	t = 4.24 *P =*0.001*
Interincisal angle	118.02 ± 8.26	115.82 ± 9.24	-2.25 (-6.33‒1.83)	t = 1.175 *P =*0.258
Wits appraisal (mm)	4.01 ± 2.26	1.71 ± 1.34	-2.55 (-3.44‒ -1.66)	t = 6.28 *P* ≤ 0.001*
Over jet (mm)	7.09 ± 1.70	4.14 ± 1.11	-2.95 (-3.63‒ -2.27)	t = 9.28 *P* ≤ 0.001*
Over bite (mm)	2.92 ± 1.44	2.29 ± 1.28	-1.41 (-2.75‒ -0.07)	t = 2.43 *P =*0.041*

* Statistically significant.

###  Facial height measurement

 A significant increase in all the measurements was observed (*P* < 0.05; Table 9).

**Table 9:** Comparison of changes in facial height measurements before and after treatment

**Facial height measurement**	**Before (n=16)**	**After (n=16)**	**Mean difference (95% CI)**	***P*** ** value**
PFH (mm)	63.92 ± 4.20	65.42 ± 3.96	1.50 (0.11‒2.9)	t = 2.29 *P* = 0.037*
TAFH (mm)	99.55 ± 5.71	103.22 ± 5.71	3.66 (1.83‒5.48)	t = 4.28 *P* = 0.001*
LAFH (mm)	57.78 ± 5.40	60.08 ± 5.23	2.31 (1.32‒3.28)	t = 4.98 *P* ≤ 0.001*

* Statistically significant.

###  Harms 

 During the study term, no harm or unanticipated effects were noticed. Only minor discomfort was felt in the initial days after using the appliance. No patients requested a prescription for an analgesic for this condition.

## Discussion

 Various orthodontic appliances can be produced using thermoplastic materials, such as the hybrid aesthetic function appliance (HAF). The concept of the HAF appliance produced from the Biocryl sheet was based on a study by Bechir et al,^11^ who observed Biocryl sheets to be more aesthetically pleasing than acrylic resin.

 This study analyzed the skeletal and dentoalveolar effects of the HAF appliance. The study revealed an insignificant effect on the growth of the maxilla. This was shown by the small nonsignificant increase in the SNA angle and the maxillary length (ANS-PNS). This outcome was in line with those of Jena et al,^12^ Janson et al,^13^ and Showkatbakhsh et al.^14^ Thus, these results were in disagreement with the finding of Clark^15^ and O’Brien et al.^2^ This difference may be attributable to the dissimilarities in the designs of the appliances, as the HAF appliance was not intended to restrict the growth of the maxilla as it did not have any component that aids in maxillary restriction such as short labial parts.

 Concerning the mandible, both the SNB angle and total mandibular length (Go-Gn) increased significantly due to the adaptive reaction to the mandibular advancement, which may result from condylar growth stimulation. The mandible’s forward position caused tendons and muscle fibers to stretch and lengthen, which in turn pulled on the muscular attachments at the surface of the bone and stimulated bone remodeling processes. Also, these findings were aligned with those of Basciftci et al^16^ and Khoja et al.^17^

 The relationship between maxillary and mandibular growth is an important factor. The ANB angle is used to measure the skeletal malocclusion in the anteroposterior dimension. The HAF appliance showed a significant reduction in ANB angle due to an increase in SNB angle and a significant decrease in the Wits appraisal by forward positioning of the mandible. These findings were in agreement with the results reported by Toth et al,^18^ Freeman et al,^19^ Trenouth^20^ and Illing et al,^21^ but different from Tümer and Gültan’s findings,^22^ as it might be related to the non-compliance of the patients leading to a nonsignificant change in the ANB angle.

 Considering the vertical dimension, this study demonstrated that the lower anterior facial height, total facial height, and posterior facial height significantly increased, which could result from the over-eruption of lower posterior teeth, as the HAF appliance did not cover the occlusal surfaces of lower posterior teeth. In contrast, angular vertical measurements (FMAº and SN/MPº) revealed no statistically significant changes, which might be attributed to increases in lower anterior facial height with an increase in posterior facial height, preventing the change in mandibular plane angle. These findings were aligned with those of Clark,^23^ Fareen et al,^24^ and Baysal and Uysal.^25^

 Concerning the dentoalveolar changes in maxillary incisors, this study also showed an insignificant change in upper incisors inclination (U1/SNº) attributable to a lack of parts that can change the inclination of teeth, such as short labial parts. This finding disagreed with Baysal & Uysal,^25^ and Patil et al^26^ due to differences in the designs of the appliances. Also, the distance from the incisal edge of the maxillary incisor to the palatal plane (U1-PP) showed an insignificant change.

 Concerning the dentoalveolar changes in mandibular incisors, labial tipping of the lower incisors was observed in this study as a significant increase in (L1/MPº), which may occur due to taking support from the lower six anterior teeth during mandibular advancement by covering the lower six anterior teeth with thermoplastic material, so forces exerted on it cause labial tipping. This finding was in agreement with Cremonini et al^27^ and Siara-Olds et al.^28^

 There was also a significant increase in distance between the lower first molar mesiobuccal cusp tip and mandibular plane (L6-GnGo (mm)) as extrusion of lower posterior teeth occurred due to freedom of occlusion posteriorly that helps in correcting the lower curve of Spee and deep bite. This result was in line with Lagerström et al^29^ studies. Considering the relation between the upper and lower incisors, the interincisal angle presented an insignificant decrease, which disagreed with the findings of Lv et al^30^ and Burhan and Nawaya.^31^

 The current study demonstrated a highly significant reduction in the overjet caused by skeletal alterations (the mandibular advancement) and dentoalveolar alteration (the lower incisor proclination). This finding coincided with the findings concerning New Clear Functional Appliance.^27^ The overbite also showed a significant reduction due to the overeruption of lower posterior teeth, according to Mills and McCulloch.^32^ Furthermore, labial tilting of lower incisors contributes to overbite reduction.

###  Recommendations

 It is advised to use a mini-implant-supported HAF appliance that reduces the dental effect and increases the skeletal effect or to extend dental coverage to lower posterior teeth that may minimize the dental effect to prevent labial tipping of lower incisors in the future newly developed version of the HAF appliance. The HAF appliance effect was not followed over a long period, which requires further investigation. Additionally, further studies are necessary to compare the dentoalveolar and skeletal effects of HAF appliances to those of conventional functional appliances.

## Limitations

 The HAF appliance is not favorable to use in high-angle patients because, in some cases, there was evidence of anterior open bite (0.5‒1 mm) during treatment. This problem was solved using the posterior bite plane at the end of the treatment.

## Conclusions

 The HAF appliance could be used to treat Cl II division 1 malocclusion. Patients accept it because it is aesthetically pleasing, comfortable, and effective. It had a skeletal effect on the growth of the mandible as it enhanced the growth of the mandibular base and mandibular length. At the same time, it affected the growth pattern of the face by increasing all facial heights. The HAF appliance improved the maxillo-mandibular relationship and reduced overjet and overbite. The HAF appliance caused significant proclination of lower incisors.

## Competing Interests

 The authors declare that they have no competing interests. The authors do not have any financial interests in the companies whose materials were included in this article.

## Ethical Approval

 The Ethics Committee of Mansoura University approved this study’s protocol (2021) under the code A05051021.

## Funding

 Funding institutions in the public, commercial, or nonprofit sectors did not provide a specific grant for this research.
